# Expression of the Hutchinson-Gilford Progeria Mutation Leads to Aberrant Dentin Formation

**DOI:** 10.1038/s41598-018-33764-6

**Published:** 2018-10-18

**Authors:** Hwajung Choi, Tak-Heun Kim, Ju-Kyeong Jeong, Charlotte Strandgren, Maria Eriksson, Eui-Sic Cho

**Affiliations:** 1 Cluster for Craniofacial Development and Regeneration Research, Institute of Oral Biosciences, Chonbuk National University School of Dentistry, Jeonju, 54896 South Korea; 2 Department of Biosciences and Nutrition, Center for Innovative Medicine, Karolinska Institutet, Huddinge, SE-14183 Sweden

## Abstract

Hutchinson-Gilford progeria syndrome (HGPS) is a rare accelerated senescence disease, manifesting dental abnormalities and several symptoms suggestive of premature aging. Although irregular secondary dentin formation in HGPS patients has been reported, pathological mechanisms underlying aberrant dentin formation remain undefined. In this study, we analyzed the mandibular molars of a tissue-specific mouse model that overexpresses the most common HGPS mutation (*LMNA*, c.1824C > T, p.G608G) in odontoblasts. In the molars of HGPS mutant mice at postnatal week 13, targeted expression of the HGPS mutation in odontoblasts results in excessive dentin formation and pulp obliteration. Circumpulpal dentin of HGPS mutants was clearly distinguished from secondary dentin of wild-type (WT) littermates and its mantle dentin by considering the irregular porous structure and loss of dentinal tubules. However, the dentin was significantly thinner in the molars of HGPS mutants at postnatal weeks 3 and 5 than in those of WT mice. *In vitro* analyses using MDPC-23, a mouse odontoblastic cell line, showed cellular senescence, defects of signaling pathways and consequential downregulation of matrix protein expression in progerin-expressing odontoblasts. These results indicate that expression of the HGPS mutation in odontoblasts disturbs physiological secondary dentin formation. In addition, progerin-expressing odontoblasts secrete paracrine factors that can stimulate odontogenic differentiation of dental pulp cells. Taken together, our results suggest that the aberrant circumpulpal dentin of HGPS mutants results from defects in physiological secondary dentin formation and consequential pathologic response stimulated by paracrine factors from neighboring progerin-expressing odontoblasts.

## Introduction

Hutchinson-Gilford Progeria Syndrome (HGPS, progeria, OMIM#176670) is a rare genetic disorder characterized by accelerated senescence^1^. Patients appear normal at birth, but during the first few years of life, symptoms start appearing and include severe growth retardation, skeletal abnormalities, delayed tooth eruption, and formation of irregular secondary dentin obliterating the dental pulp^2–4^. The HGPS phenotype is the consequence of accumulation of progerin, a mutant form of lamin A. Lamin A is localized to the nuclear lamina at the inner-side of the nuclear envelope, contributing to nuclear structural stability and other nuclear functions^5^. The mutation causing progerin expression is a recurrent, de novo, dominant point mutation (c.1824C > T) in the *LMNA* gene that leads to a 50-residue truncation of the lamin A protein^6,7^. This truncated lamin A retains the CAAX motif but lacks the second proteolytic cleavage site of ZMPSTE24, an endoprotease responsible for the final cleavage of prelamin A into mature lamin A^8,9^. The cellular defects in HGPS stem from the accumulation of progerin, which leads to nuclear membrane distortion and a decreased cellular life span. Skeletal defects may be secondary to accumulation of progerin within skeletal tissue^10^. HGPS is a disease with major phenotypic features of accelerated cellular, physiological, and anatomical aging of most major tissues and organs^11^.

Dentin is produced by odontoblasts, specifically differentiated cells derived from ectomesenchyme^12^. Unlike other cells derived from mesenchyme, molar odontoblasts exhibit unique features such as a well-formed junctional complex, and no replacement with newly differentiated odontoblasts as they are terminally differentiated in the physiologic state^13^. Three types of dentin have been identified in teeth: primary dentin, which forms rapidly, in association with enamel or cementum apposition, during tooth formation; secondary dentin, which results from the continued but relatively slow apposition of dentin in later life and may be associated with a reduction in the number of functioning odontoblasts^13^; and tertiary dentin, laid down as a localized response to trauma^14^. Pulp tissue responds to dentin damage by laying down a tertiary dentin matrix (reactionary or reparative) beneath the site of injury. Generally, tertiary dentin is divided by the microstructure of the mass: when pulp cells secrete dentin, the resulting material has an amorphous, atubular, calcified tissue structure referred to as fibrodentin; however, when preexisting odontoblasts secrete dentin, the resulting material has a tubular structure^15,16^.

Previous results showed that tissue-specific inducible expression of human lamin A, carrying the HGPS mutation, during skeletal development in mice caused bone abnormalities with poor biomechanical properties and widespread loss of osteocytes and osteoblasts^17^. It was also reported that expression of the HGPS mutation during tooth development resulted in irregular dentin formation and polarization defects in the same mutant mouse strain^17^. However, it remains to be clarified how the HGPS mutation operates in the process of dentin formation during tooth development. In this study, different age groups of transgenic mice with tissue-specific expression of the HGPS mutation were histologically analyzed, and in addition *in vitro* techniques were used to uncover the underlying molecular mechanisms following expression of the HGPS mutation in odontoblasts.

## Results

### Expression of the HGPS mutation in odontoblasts results in excessive dentin formation and pulp obliteration

**Figure 1:**
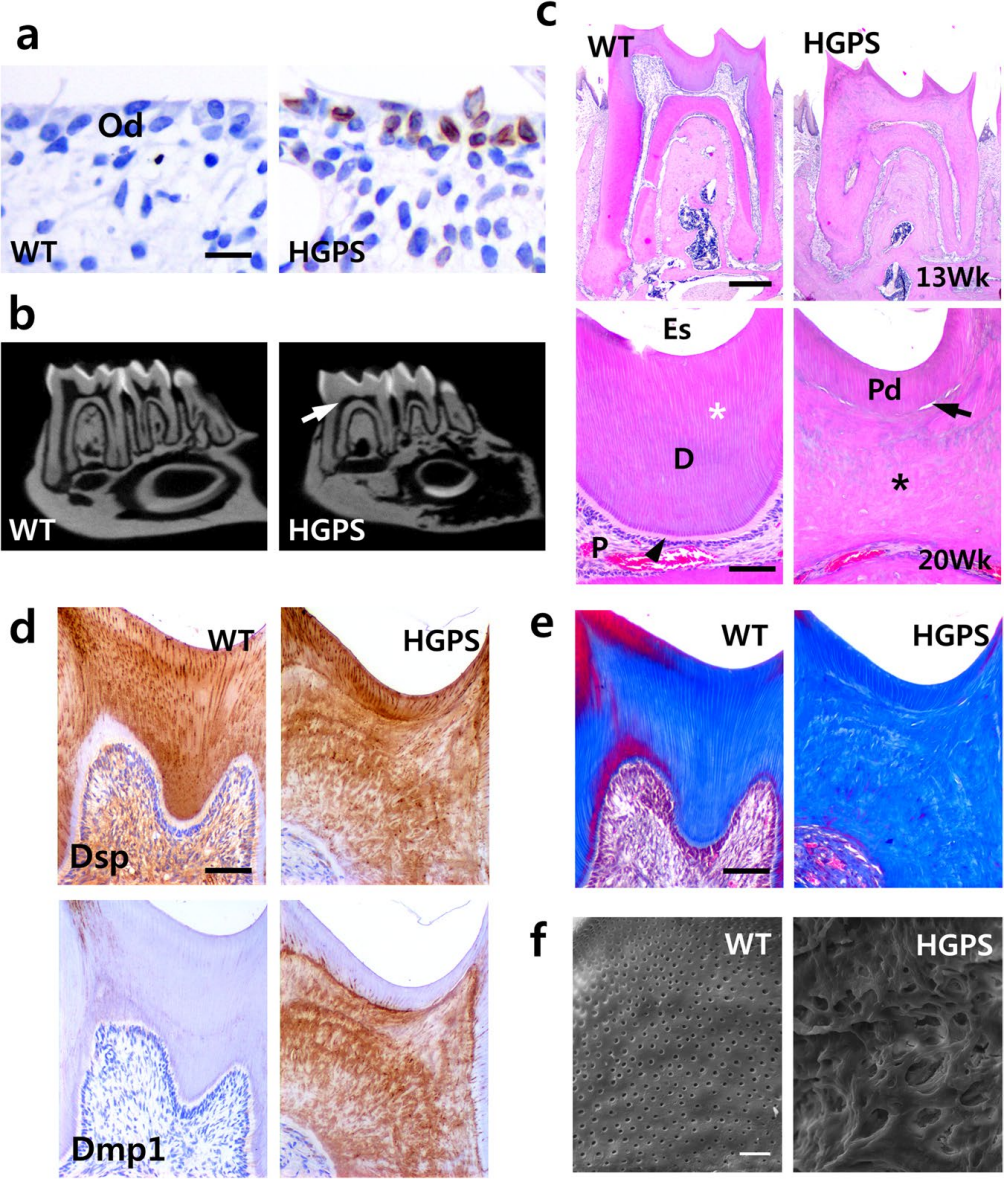
Expression of the HGPS mutation in odontoblasts results in excessive dentin formation and pulp obliteration. (**a**) Transgene expression in the odontoblasts of HGPS mice at postnatal week 13 was exhibited by immunohistochemistry with mouse mandibular tissue sections using the anti-human lamin A + C antibody. (**b**) Excessive dentin formation and the consequential pulp obliteration (white arrow) were exhibited by micro-computed tomographic view of mandibles of HGPS mice at postnatal week 13. (**c**) H&E-stained images of mandibles from WT and HGPS mice at postnatal week 13 (top) and week 20 (bottom) showing pulp obliteration (black asterisk) caused by irregular excessive dentin formation. In contrast to the continuous dentinal tubules (white asterisk) present in the WT mice, newly deposited dentin was not continuous with primary dentin in HGPS mice and could be distinguished by a demarcation line (arrow) between primary and irregular excessive dentin. Predentin (black arrow head) was well formed underneath preexisting dentin in WT but not in HGPS mice. (**d**) Immunohistochemistry was performed by using the anti-Dsp antibody. Dsp was localized in the dentin of both WT and HGPS mice, except in the predentin layer in WT mice (top). Dmp1 was highly expressed only in newly formed irregular excessive dentin of HGPS mice (bottom). (**e**) Confirmation of histological differences in coronal dentin by trichrome staining of mouse molar at postnatal week 13. (**f**) Microstructure of newly formed dentin by scanning electron microscopy showed aberrant dentin in HGPS mice at postnatal week 13. Newly formed dentin in HGPS mice was irregular and porous, whereas dentinal tubules were regularly aligned in the secondary dentin of WT mice. Od, odontoblasts; D, dentin; Es, enamel space; P, pulp; Pd, primary dentin. *Bars*, 20 μm (**a**), 100 μm (**c** bottom, **d**, **e**), 400 μm (**c** top, **f**).

To understand the influence of the HGPS mutation on dentin formation, we characterized the dental phenotype of transgenic mice with tissue-specific expression of HGPS mutation in odontoblasts. As with human teeth of patients carrying the HGPS mutation, the most remarkable feature of the adult molars in HGPS mutant mice was pulp obliteration caused by irregular excessive dentin formation^2^. To confirm HGPS transgenic expression in adult mice, we performed immunohistochemistry with dental tissues from wild-type (WT) and HGPS mice at postnatal week 13 using an anti-human lamin A + C monoclonal antibody (Fig. 1a). Transgenic expression was observed only in the odontoblasts of HGPS mutants; no immunoreactivity was observed in WT mice. Micro-computed tomography (Micro-CT) revealed that the dentin thickness was significantly greater in the crowns of HGPS mutant molars than in those of WT mice at postnatal week 13 (Fig. 1b). Likewise, observation of the hematoxylin and eosin (H&E)-stained sections also indicated that the dentin thickness of the HGPS mutant molars was greater than that of the WT mice molars at postnatal week 13 (Fig. 1c top). Furthermore, most of the pulp chamber and root canals were filled with excessive dentin deposition in HGPS mutants at postnatal week 20 (Fig. 1c bottom). In contrast to the continuous dentinal tubules present in the WT mice, newly deposited dentin was not continuous with primary dentin in HGPS mice and could be distinguished by a demarcation line between primary and secondary dentin.

The immunohistochemical analysis showed that dentin sialoprotein (Dsp), a major noncollagenous protein in dentin, was localized in the primary and secondary dentin, except predentin, in WT mice at postnatal week 13 (Fig. 1d). In HGPS mutant mice, Dsp was widely localized in the dentin matrix of primary and secondary dentin. There was a large difference in the expression of dentin matrix protein 1 (Dmp1) between WT mice and HGPS mutants. While weak or rare expression of Dmp1 was localized in the dentin of WT mice, extensive expression of Dmp1 occurred in secondary dentin of HGPS mutants but not primary dentin (Fig. 1d). Trichrome staining of mouse molars at postnatal week 13 confirmed the histological differences in coronal dentin between WT mice and HGPS mutants (Fig. 1e). The microstructure of new dentin is closely related with the cellular source of its mass^15,16^. To assume cellular source of the mass in HGPS mutant mice, we compared the microstructure of secondary dentin in WT mice and HGPS mutant mice at postnatal week 13 using acid etched scanning electron microscope (SEM). The secondary dentin of HGPS mutants was a porous, bone-like, atubular, calcified tissue generally formed by pulp cells, while that of WT mice showed robust, regular tubular structures formed by odontoblasts (Fig. 1f). These results imply that the secondary dentin of HGPS mutants might be formed in response to cellular injury following expression of the HGPS mutation in odontoblasts.

### Expressing the HGPS mutation in odontoblast disturbs physiological secondary dentin formation

**Figure 2:**
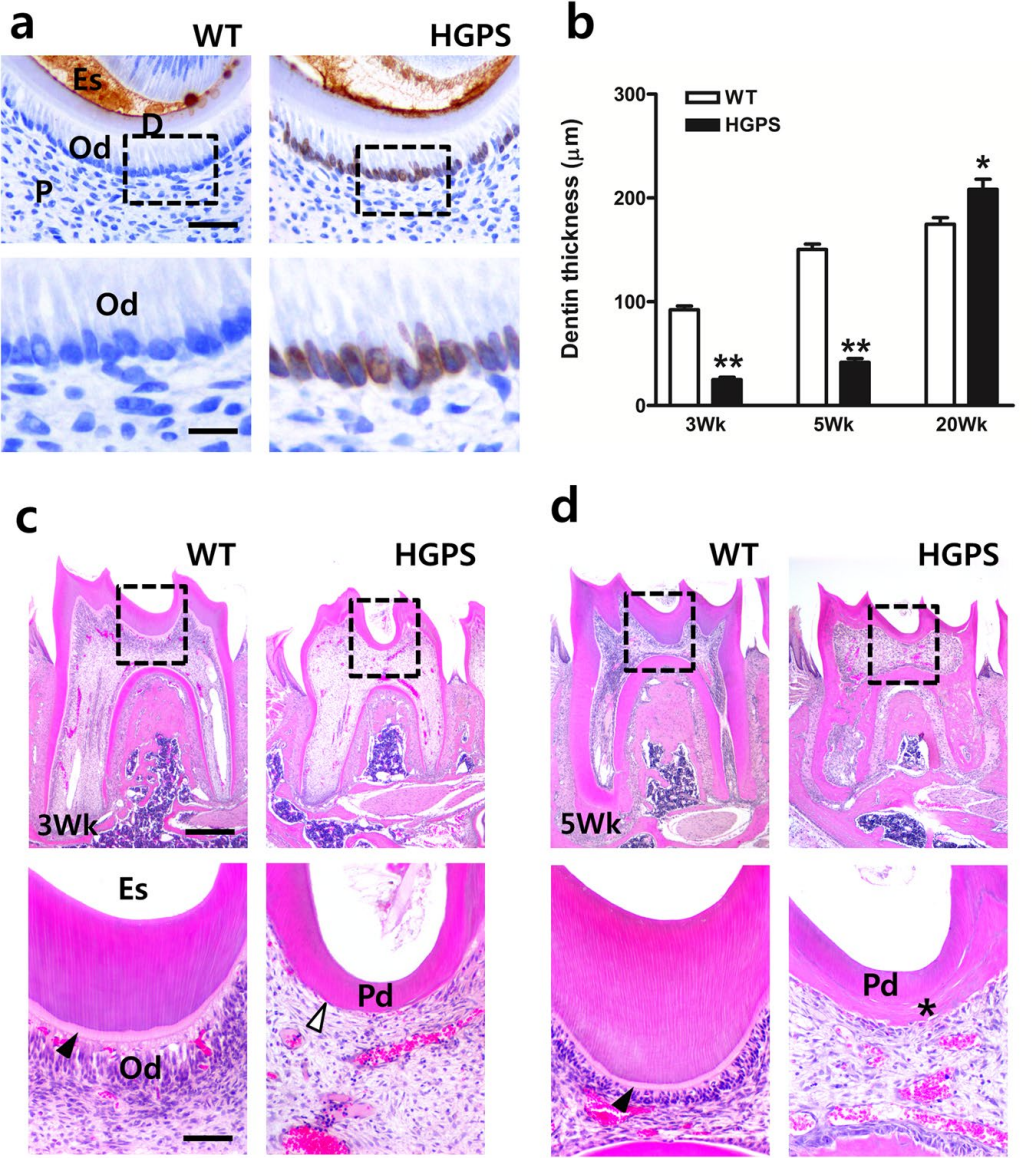
Expression of the HGPS mutation disturbs physiological secondary dentin formation. (**a**) Transgenic expression in the odontoblasts of HGPS mice at postnatal day 5. Bottom images are enlarged from the boxed area of the top images. (**b**) Differences in dentin thickness of WT and HGPS mice at postnatal week 3, 5, and 20 (n = 3–5, in each genotype and age group, respectively). *p < 0.05 and **p < 0.01. (**c**) Impairment of secondary dentin formation and loss of predentin layer (empty arrow head) in the molar of HGPS mutants at postnatal week 3. Predentin (black arrow head) was well formed underneath preexisting dentin in WT mice. Bottom images are enlarged from the boxed areas of the top images. (**d**) Apposition of aberrant dentin (black asterisk) beneath the primary dentin in HGPS mutants at postnatal week 5. Bottom images are enlarged from the boxed areas of top images. Od, odontoblasts; D, dentin; Es, enamel space; P, pulp; Pd, primary dentin. *Bars*, 20 μm (**a** bottom), 40 μm (**a** top), 100 μm (**c** bottom), 400 μm (**c** top).

To get insight into the mechanical processes for irregular secondary dentin formation in HGPS mutants, we compared coronal dentin formation of WT mice and HGPS mutants by age. Transgenic expression of the HGPS mutation could be confirmed in HGPS mutant mice already at postnatal day 5, with odontoblasts still displaying intact morphology despite expressing the transgene, whereas WT mice showed no immunoreactivity (Fig. 2a). Interestingly, at younger ages such as postnatal weeks 3 and 5, the dentin thickness in HGPS mice was dramatically reduced compared to WT mice, which was an inversion of the situation in the coronal dentin thickness between HGPS and WT mice at postnatal week 20 (Fig. 2b). The remarkable differences in dentin thickness between HGPS and WT mice were also visualized using H&E-stained sections (Fig. 2c,d). The decrease in the coronal dentin thickness was more evident in HGPS mutants at postnatal week 5 than week 3 (Fig. 2d). The odontoblasts seemed to have disappeared, with no predentin in HGPS mutant mice in either age group, while the odontoblasts in WT mice appeared as a tall columnar shape in multiple aligned layers located underneath the predentin layer. In addition, the dentinal tubules of the secondary dentin in HGPS mutants were impaired, whereas the robust dentinal tubules of WT mice were continuous at postnatal week 5 (Fig. 2d bottom). These results indicate that primary dentin formation was not affected, but secondary dentin formation was impaired in HGPS mutant mice, implying that each dentin layer of HGPS mutant mice was differently affected by progerin expression.

### Induction of DNA damage and cellular senescence by progerin expression in odontoblasts

**Figure 3:**
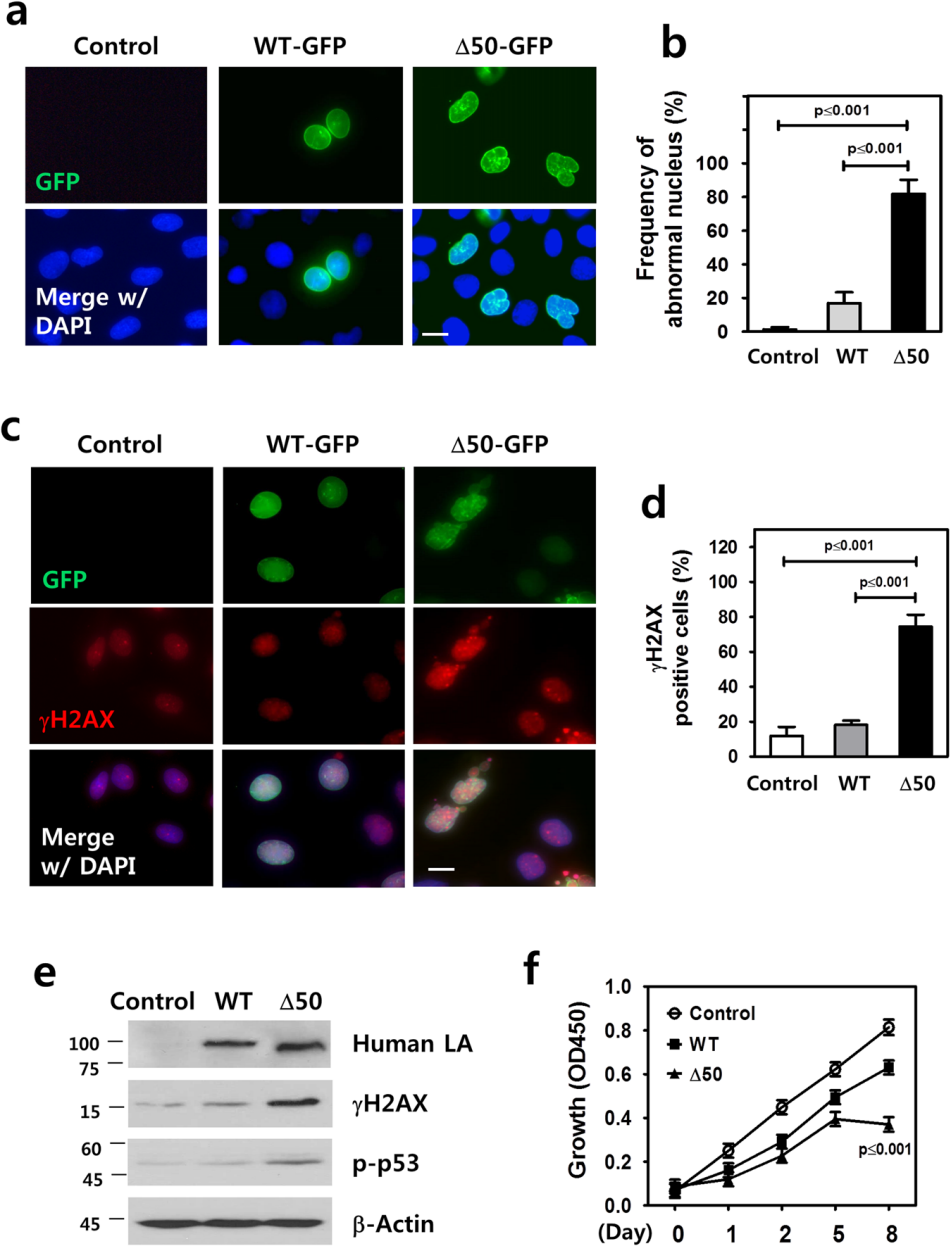
Induction of DNA damage and cellular senescence by progerin expression in odontoblasts. (**a**) Progerin-elicited structural changes in the nuclear envelope of MDPC-23 cells with Δ50 lamin A were compared to the WT lamin A and the negative control. (**b**) Percentage of cells with abnormal nucleus was compared by scoring blebs or invaginations along the membrane. (**c**,**d**) Phosphorylated H2AX-positive nuclei in MDPC-23 with Δ50 lamin A were detected by immunofluorescence staining using antibodies for γH2AX (**c**) and their counted percentage (**d**) compared to the WT lamin A and the negative control. (**e**) Confirmation of increased DNA damage in MDPC-23 with Δ50 lamin A by Western blot analysis using antibodies for human lamin A (LA), γH2AX and phosphorylated p53 (p-p53). The samples shown were derived from the same experiment, and all gels/blots were processed under the same experimental conditions. β-actin was used as a loading control. Cropped images are displayed here; the original full-size blots are presented in Supplementary Fig. S5. (**f**) Growth rates of MDPC-23 cells with Δ50 lamin A, WT lamin A and the negative control were compared until day 8 in serum-free media. Significance was assigned for *p*-values as indicated. *Bars*, 5 μm (**a** and **c**).

To understand the molecular mechanism of expressing the HGPS mutation in odontoblasts during dentinogenesis, we used MDPC-23 cells for *in vitro* analyses. To evaluate progerin-elicited structural changes in the nuclear envelope or indirect DNA damage, we performed immunofluorescence staining in MDPC-23 cells after transfection of genes encoding human WT lamin A and its HGPS mutant form (Δ50) expressing truncated lamin A, progerin. Progerin expression in MDPC-23 cells resulted in nuclear blebs and invaginations, demonstrating decreased structural integrity of the nuclear membrane, as previously reported^6^. Most of the nuclei in MDPC-23 cells with the expression of Δ50 lamin A were shaped abnormally, whereas cells with expression of WT lamin A mostly had robust, round-shaped nuclei (Fig. 3a). The nuclei were scored as abnormal if they contained one or more blebs or invaginations along the membrane. A significantly higher percentage of cells with abnormal nuclei were detected in MDPC-23 with Δ50 lamin A (81.88 ± 16.75%) compared to WT lamin A (16.96 ± 12.84%) and the negative control (1.32 ± 2.63%) after selection by antibiotics (Fig. 3b, *p* ≤ 0.001). As defective maintenance of genomic integrity exists in laminopathy-based premature aging, we asked whether DNA damage occurred in odontoblasts harboring the HGPS mutation. Upon DNA damage, histone H2AX is phosphorylated at sites of DNA lesions (γH2AX), which triggers a DNA damage checkpoint response^18^. Using immunofluorescence staining, we observed more γH2AX-positive nuclei in MDPC-23 with Δ50 lamin A compared to the WT lamin A control (Fig. 3c), indicating greater DNA damage in cells with progerin expression. By quantification of γH2AX-positive nuclei we found a significantly higher percentage of cells with numerous γH2AX foci in MDPC-23 with Δ50 lamin A (74.42 ± 15.27%) compared to those observed in WT lamin A (18.13 ± 5.47%) and the negative control (11.85 ± 8.98%), implicating progerin-induced DNA damage signaling (Fig. 3d, *p* ≤ 0.001). Phosphorylation of p53, a hallmark of DNA damage-induced responses activated by ATM (ataxia-telangiectasia mutated) and ATR (ATM- and Rad3-Related) kinases^19^, was also increased in MDPC-23 with Δ50 lamin A compared to the WT lamin A control (Supplementary Fig. S1, *p* ≤ 0.001). Increased DNA damage was further confirmed by Western blotting, which showed elevated expression of γH2AX and phosphorylated p53 (p-p53) in MDPC-23 cells with Δ50 lamin A (Fig. 3e). Human lamin A was also detected by Western blotting using an anti-human lamin A + C monolclonal antibody (MAB3211, Chemicon) that binds to both human WT lamin A and progerin proteins. This antibody does not cross-react with mouse lamin A and C. We investigated whether the cellular growth of Δ50 lamin A-expressing odontoblasts was affected by comparison of proliferation rates of MDPC-23 cells with Δ50 lamin A, WT lamin A and the negative control. The overexpression of WT lamin A slightly reduced the growth rate of MDPC-23 cells compared to the control at day 8, but compared to WT lamin A the cells expressing Δ50 lamin A decreased significantly at the same day (Fig. 3f, *p* ≤ 0.001).

### Defects in signaling pathways and downregulation of matrix protein expression in progerin-expressing odontoblasts

**Figure 4:**
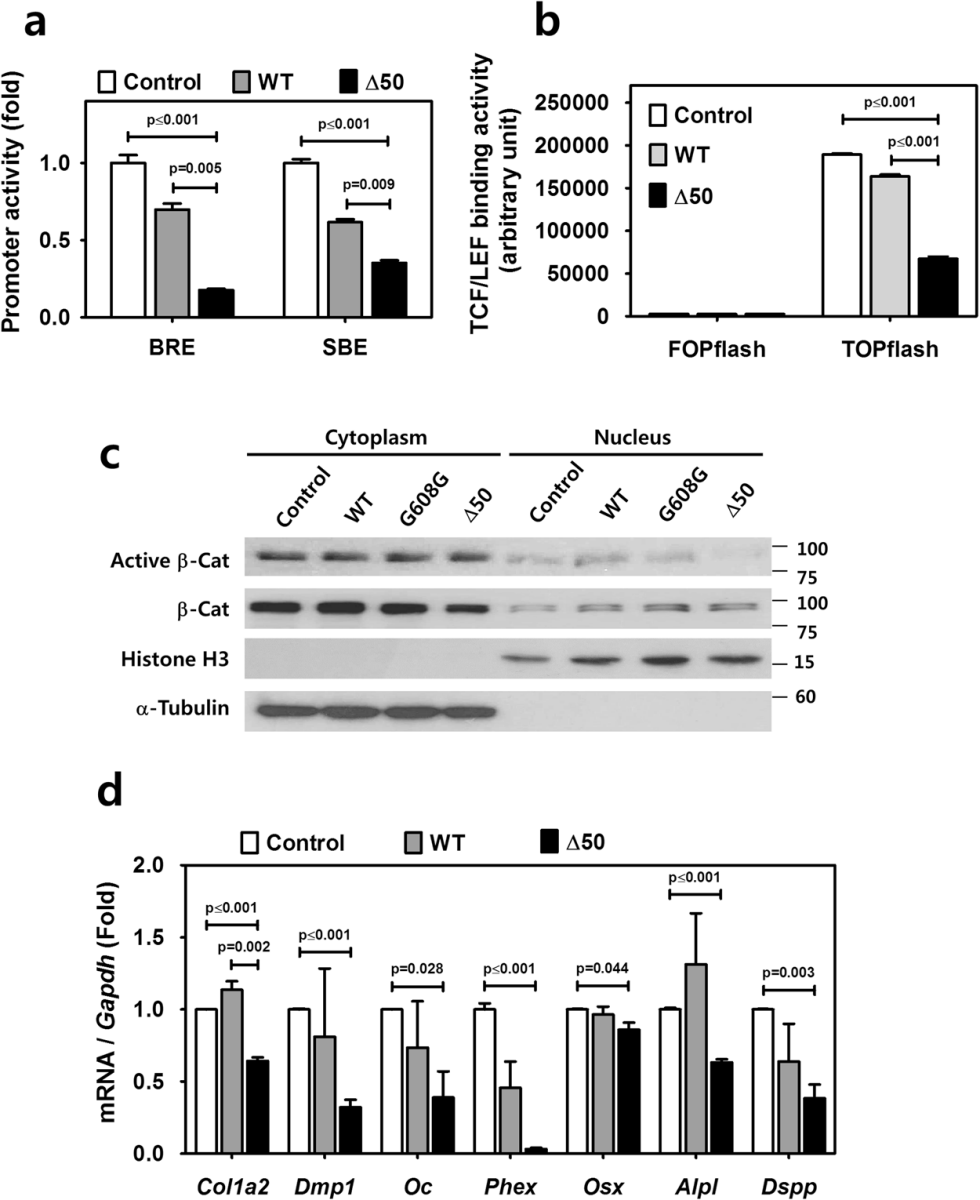
Defects in signaling pathways and downregulation of matrix protein expression in progerin-expressing odontoblasts. (**a**) Promoter activities for BMP and TGFβ signaling were analyzed and compared in MDPC-23 transfected with Δ50 lamin A, WT lamin A and the negative control by luciferase reporters of BRE and SBE, respectively. (**b**) Promoter activities for Wnt/β-catenin signaling were analyzed and compared in MDPC-23 transfected with Δ50 lamin A, WT lamin A and the negative control by luciferase reporters of FOPflash/TOPflash. (**c**) Western blot of cytoplasmic and nuclear protein was performed using antibodies for nonphosphor-β-catenin (Active β-Cat), β-catenin (β-Cat), histone H3 and α-tubulin. Progerin expression by transfection of G608G lamin A and Δ50 lamin A impairs nuclear translocation of β-catenin. The samples shown were derived from the same experiment, and all gels/blots were processed under the same experimental conditions. α-tubulin and histone H3 were used as a loading control for cytoplasmic and nuclear protein, respectively. Cropped images are displayed here; the original full-size blots are presented in Supplementary Fig. S6. (**d**) The transcript levels of dentin matrix-related genes in MDPC-23 transfected with Δ50 lamin A, WT lamin A and the negative control were analyzed and compared by real-time qPCR. Significance was assigned for *p*-values as indicated.

Abnormalities in the nuclear envelope can lead to alterations in cell signaling pathways that underlie development^20^. Tooth development including odontogenesis features a sophisticated series of signaling interactions^21–23^. To test the possibility of alterations in cell signaling pathways that underlie dentin formation, we investigated whether the promoter activities for BMP, TGF-β and Wnt/β-catenin signaling were affected using luciferase reporters. As shown in Fig. 4a,b, the promoter activities for BMP, TGF-β and Wnt/β-catenin signaling were significantly reduced by the expression of Δ50 lamin A in MDPC-23 when compared to WT and the negative control as seen by luciferase reporters of BMP response element (BRE), Smad binding element (SBE) and FOPflash/TOPflash, respectively (*p*-values are indicated in the figures). The reporter of BRE reflects the transcriptional activation of Id1 transcripts, which is one of several genes directly targeted by BMP signaling. The Smad complex is a messenger of TGFβ and binds to the SBE in the nucleus, leading to transcription and expression of TGFβ/Smad responsive genes. TOPflash holds wild-type TCF binding regions to activate transcription of Wnt target genes, whereas FOPflash holds mutated TCF binding regions, which is used as the negative control. We have previously reported that Wnt/β-catenin signaling plays a critical role in the differentiation of odontoblasts and secretion of matrix protein in dentin formation^23,24^. To provide a more detailed analysis of the reduced β-catenin transcriptional activity with progerin expression and also in the presence of lamin A expression, since this is what is happening in the HGPS patients, we transfected the G608G lamin A (that express both progerin and lamin A) and the Δ50 lamin A (that only express progerin) expression vectors. The results showed that progerin expression impairs nuclear translocation of β-catenin. As shown in Fig. 4c, both progerin-expressing cells have reduced amounts of nuclear β-catenin, which is a sign of impairment in nuclear translocation of β-catenin. The impairment in nuclear translocation of active β-catenin was also visualized by immunofluorescence staining of non-phosphorylated β-catenin (S33/37/Thr41) chemically activated by Wnt agonist 1, a Wnt signaling pathway activator (Supplementary Fig. S2). These results all imply that progerin might affect nuclear translocation of β-catenin by nuclear envelope alteration, as suggested previously^20^. These impairments of signaling pathways were associated with downregulated mRNA levels of a panel of dentin matrix-related genes. Collagen 1a2 (*Col1a2*) transcript was significantly reduced and other genes including dentin matrix protein 1 (*Dmp1*), osteocalcin (*Oc*), phosphate-regulating gene with homologies to endopeptidases on the X-chromosome (*Phex*), osterix (*Osx*, *Sp7*), tissue non-specific alkaline phosphatase (*Alpl*) and dentin sialophosphoprotein (*Dspp*) showed a tendency toward lower expression when compared to WT lamin A (Fig. 4d, *p*-values are indicated in the figure).

### Paracrine factors induced by progerin stimulate odontogenic differentiation of dental pulp cells

**Figure 5:**
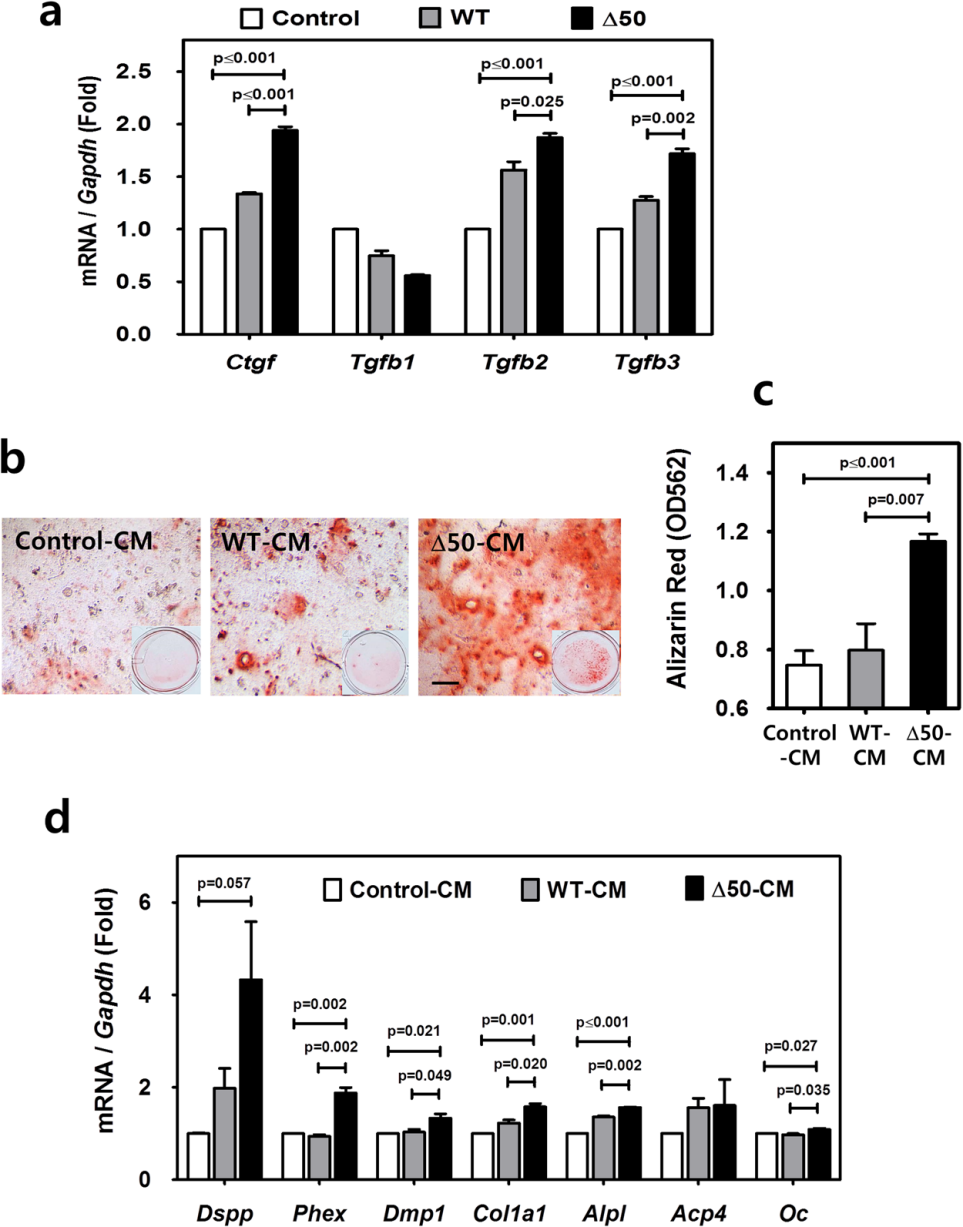
Paracrine factors induced by progerin stimulate odontogenic differentiation of dental pulp cells. (**a**) The transcript levels of genes for soluble factors were analyzed by real-time qPCR of MDPC-23 transfected with Δ50 lamin A, WT lamin A and the negative control. (**b**,**c**) Mineralization ability of dental pulp cells treated with conditioned media from MDPC-23 cells of Δ50 lamin A (Δ50-CM), WT lamin A (WT-CM) and the negative control (Control-CM) was exhibited by alizarin red staining (**b**) and evaluated (**c**). (**d**) The transcript levels of odontogenesis-related genes were analyzed by real-time qPCR with dental pulp cells treated with conditioned media of Δ50 lamin A (Δ50-CM), WT lamin A (WT-CM) and the negative control (Control-CM) for 2 days. Significance was assigned for *p*-values as indicated. *Bars*, 40 μm (**b**).

**Figure 6:**
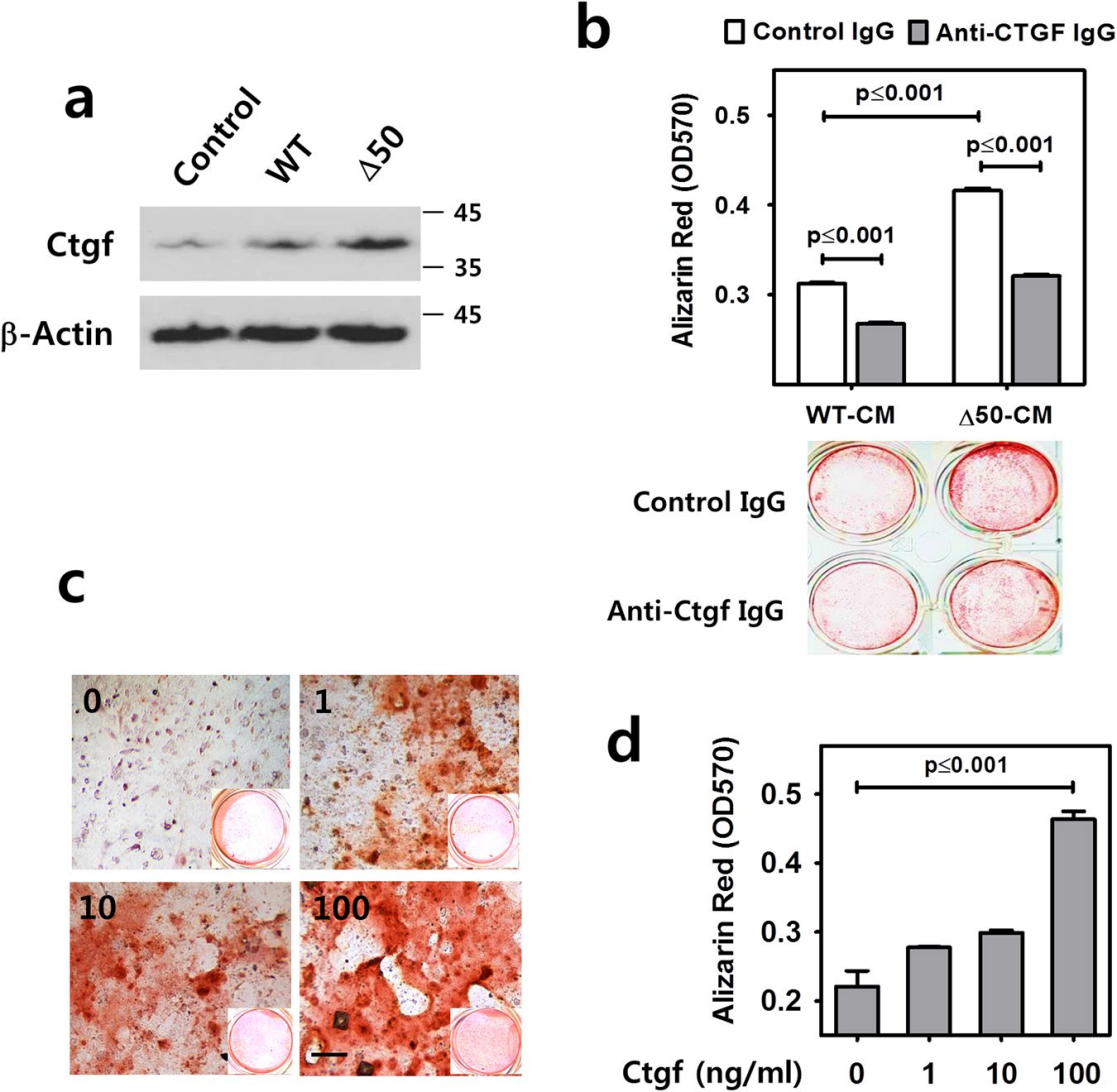
Ctgf induced by progerin stimulates odontogenic differentiation of dental pulp cells. (**a**) The expression of Ctgf in odontoblasts was analyzed with whole cell extract from MDPC-23 transfected with Δ50 lamin A, WT lamin A and the negative control by Western blot analysis using an antibody for Ctgf. The samples shown were derived from the same experiment, and all gels/blots were processed under the same experimental conditions. β-actin was used as a loading control. Cropped images are displayed here; the original full-size blots are provided in Supplementary Fig. S7. (**b**) Reduced mineralization of mouse dental pulp cells by neutralization of Ctgf using polyclonal anti-Ctgf IgG. Alizarin red staining was performed with mouse dental pulp cells treated with conditioned media from the culture of MDPC-23 cells expressing Δ50 lamin A (Δ50-CM) and WT lamin A (WT-CM) supplemented with polyclonal anti-Ctgf IgG (2 μg/ml) and control IgG for 2 days. (**c**,**d**) Mineralization ability of dental pulp cells treated with different amounts of recombinant Ctgf peptide was exhibited by alizarin red staining (**c**) and evaluated (**d**). Significance was assigned for *p*-values as indicated. *Bars*, 40 μm (**c**).

Previous work has demonstrated that certain paracrine factors promote tissue repair locally^25,26^. We investigated whether progerin-expressing odontoblasts express dentinogenesis-related soluble factors including TGFβ family members and facilitate odontogenic differentiation of dental pulp cells through the paracrine influence. As shown in Fig. 5a, the mRNA levels of *Ctgf*, *Tgfb2* and *Tgfb3* were significantly increased by the expression of Δ50 lamin A in MDPC-23 when compared to WT and the negative control (*p*-values are indicated in the figure). However, *Tgfb1* and other members of the TGFβ family such as *Bmp2* and *Bmp4* or pro-inflammatory factors such as interleukin-6 (*Il-6*), interleukin-8 (*Il-8*), matrix metalloproteinase 3 (*Mmp3*) and tissue inhibitor of matrix metalloproteinase 1 (*Timp1*) were not significantly induced by the expression of Δ50 lamin A under the same condition (Supplementary Fig. S3). Next, to evaluate the paracrine influence of progerin expression in odontoblasts, we treated mouse dental pulp cells with conditioned media from the culture of MDPC-23 cells with HGPS mutation expression. As shown by alizarin red staining (Fig. 5b) and its subsequent quantitation (Fig. 5c), the mineralization ability of dental pulp cells was significantly increased by conditioned media of Δ50 lamin A expression when compared to that of WT and negative control (*p*-values are indicated in the figure). In addition, treatment with conditioned media of Δ50 lamin A induced the expression of odontogenesis-related genes including *Dspp*, *Phex*, *Dmp1*, Collagen 1a1 (*Col1a1*), *Alpl*, *Acp4* and *Oc* (Fig. 5d, *p*-values are indicated in the figure). Increased expression of Ctgf protein in progerin-expressing odontoblasts was also confirmed by Western blot analysis (Fig. 6a). The paracrine action tested using conditioned media from progerin-expressing odontoblasts also showed an effect on the differentiation of undifferentiated odontoblasts (Supplementary Fig. S4). In addition, to neutralize its paracrine activity, we treated polyclonal anti-Ctgf IgG to mouse dental pulp cells with conditioned media from the culture of MDPC-23 cells expressing Δ50 and WT lamin A. Polyclonal anti-Ctgf IgG treatment significantly reduced mineralization activity of mouse dental pulp cells in both WT- and Δ50 lamin A-conditioned media groups (Fig. 6b, *p* ≤ 0.001). Furthermore, we evaluated the mineralization ability of Ctgf on mouse dental pulp cells by alizarin red staining after the treatment of mouse dental pulp cells with different amounts of recombinant Ctgf peptide (Fig. 6c,d). The results suggest that Ctgf plays a major role in the effect of conditioned media to stimulate odontogenic differentiation of dental pulp cells.

## Discussion

**Figure 7:**
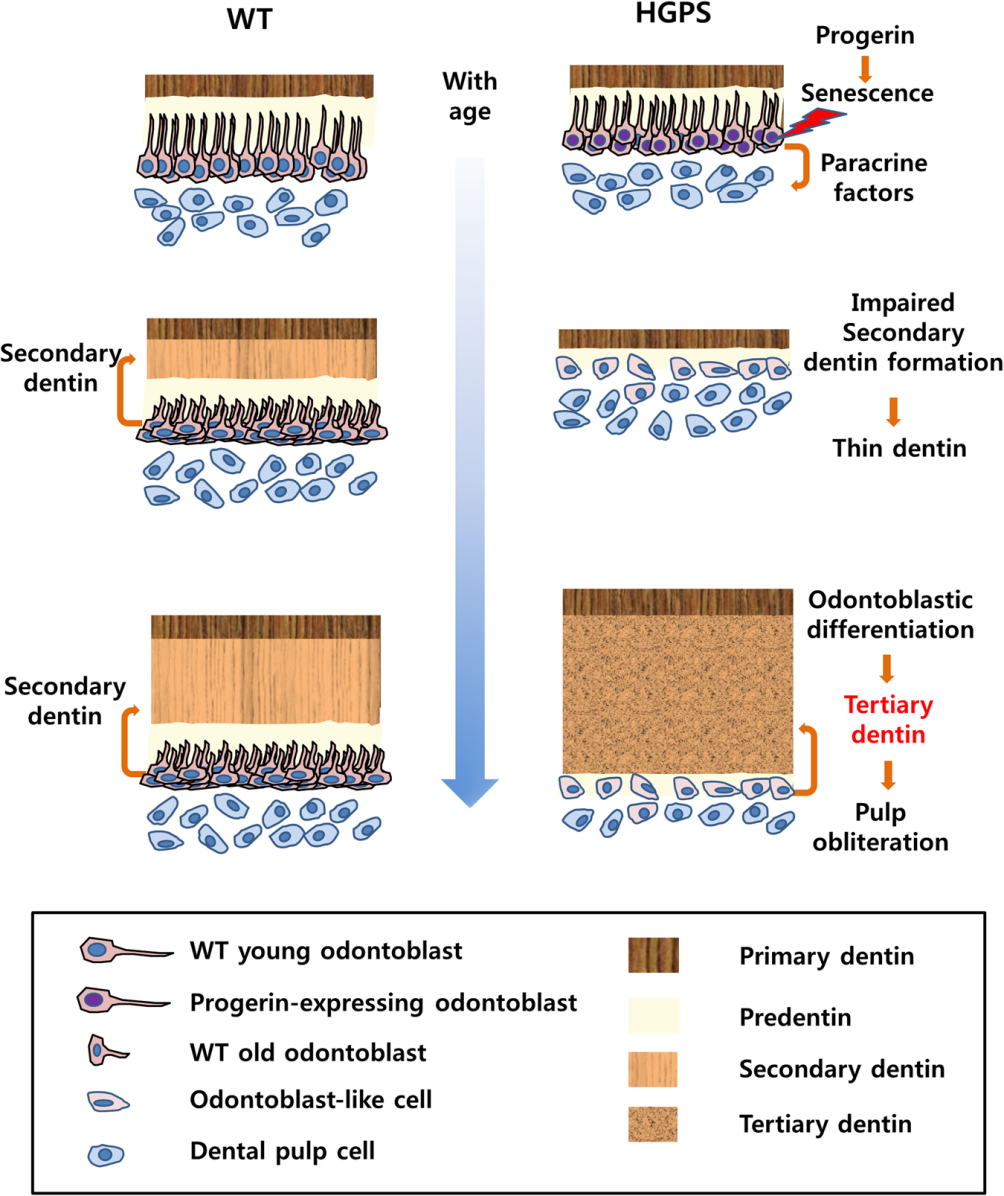
Schematic working model proposing the major hypothesis. During initial dentinogenesis in WT, odontoblasts form a primary dentin until the tooth becomes functional. When contacts between antagonistic cusps are established, then the formation of secondary dentin starts immediately, and continues throughout life. However, in HGPS mice, progerin-induced cellular senescence of odontoblasts occurred by progerin results in impaired physiological secondary dentin formation, which is an intermediate outcome. Physiologically, this thinner dentin may be recognized as an injury, such as dentin trauma. Subsequently, odontoblast-like cells differentiated from dental pulp cells might rapidly produce tertiary dentin until the pulp is completely obliterated in the HGPS mice. Paracrine factors released from odontoblasts undergoing senescence accelerate tertiary dentin formation by stimulating odontogenic differentiation of pulp cells.

The most striking feature of the teeth in HGPS patients and the skeletal tissue-specific mouse model harboring the most common HGPS mutation was the early formation of irregular secondary dentin obliterating the dental pulp^2,17^. The irregular excessive dentin of HGPS mutant mice has discontinuous dentinal tubules, unlike that of WT mice. Newly deposited dentin of HGPS mice also differs from primary dentin by its discontinuous dentinal tubules and porous structure, which is similar to tertiary dentin. In the adult molar, as seen at postnatal week 13 and 20, only secondary dentin formation was severely impaired by progerin expression, while primary dentin formation was not affected. However, in the growing molars of HGPS mutant mice at postnatal week 3 and 5, the dentin thickness was significantly thinner than in the WT littermates. Based on the chronological analysis of dental tissues from HGPS mutant mice, it appears that normal development of dentin underlies primary dentin formation, but irregular circumpulpal dentin is deposited beneath mantle dentin beginning at postnatal week 5 and gradually obliterates almost the entire pulp cavity by postnatal week 20. As seen by SEM, irregular circumpulpal dentin of HGPS mutant mice showed porous bone-like structure, unlike the regular tubular structure in its own mantle dentin and the circumpulpal dentin of WT mice. It is generally accepted that dentin produced by odontoblasts has a tubular structure, while the dentin produced by pulp cells does not^16^. The microstructure of circumpulpal dentin confirms that new dentin formation was triggered by odontoblast injury and rapidly deposited by odontoblast-like cells derived from pulp cells. The results from phenotype analysis strongly indicate that expression of the HGPS mutation in odontoblasts disturbs physiological secondary dentin formation, but promotes tertiary dentin formation (Fig. 7).

Lamin A regulates nuclear functions including DNA replication, DNA repair, chromatin and nuclear pore complex organization, and gene expression by directly binding to DNA^27,28^. Progerin expression delocalizes nuclear envelope proteins, disorganizes heterochromatin and nuclear pore complexes, disrupts nuclear morphology, and increases DNA damage and repair^5,6,27,29^. During progerin expression controlled by Sp7 (Osx) expression, dramatic changes were observed in the odontoblast phenotype of HGPS mutant mice. The odontoblasts were characterized by a reduced cell size and flattened shape, both of which are age-related changes related to senescence^30^. Senescence is a cellular response to damage characterized by cell cycle arrest and the secretion of pro-inflammatory cytokines and other tissue-repairing soluble factors with pleiotropic functions^26,31^. *In vitro* analyses using a mouse odontoblastic cell line demonstrated that progerin-expressing odontoblasts were characterized by a set of core features, including durable growth arrest, activation of DNA damage molecules such as γH2AX and p53, and expression of a number of soluble factors such as Ctgf and TGF-β. Of note, Ctgf has been noted to play an important role during dentin repair. Strong expression of Ctgf has been detected in odontoblast-like cells lining the reparative dentin subjacent to dental caries^32^.

Progerin expression also disturbs signaling pathways underlying developmental and normal physiological states by inflicting an abnormal nuclear envelope^33^. Wnt/β-catenin signaling is a key molecular pathway driving tooth organogenesis, including secondary dentin formation^23,24^. Interestingly, Wnt signaling functions couple extracellular matrix with nuclear lamina in progeria^34^. In addition, dentin injury triggered a dramatic increase in endogenous Wnt signaling, demonstrated by widespread distribution of Wnt-responsive cells after dentin injury^16^. Recently, using an animal model it has been proven that Wnt-responsive status is critical for the formation of reparative dentin as well as physiological secondary^16^. A recent study from Choi and colleagues identified impaired β-catenin signaling in HGPS during osteoblast differentiation^35^. This study, together with the results reported here, suggests that β-catenin signaling might be a therapeutic target for some of the phenotypes associated with this disease. TGF-β/ΒΜP signaling is also critical to dentin matrix formation. Tissue-specific disruption of *Bmp2* and *Bmp4* caused impairment of dentin matrix formation^36,37^. Odontoblast-specific disruption of *Smad4* using *Col1a1*-, *Osteocalcin*-, and *Dspp*-Cre resulted in gradual impairment of secondary dentin formation^38^. In addition, disruption of *Tgfbr2* in odontoblasts leads to severe impairment of secondary dentin formation with idiopathic pulpal calcification^39^. Similarly, disruption of *Bmpr1a* in odontoblasts leads to secondary dentin defects without idiopathic pulpal calcification^39^. Both these reports and our results suggest that progerin disturbs critical signaling involved in odontogenesis collectively required for proper secondary dentin formation.

Besides the effects on the microstructure of dentin in HGPS mice, the features of irreversible growth arrest and signaling disruption in progerin-expressing odontoblasts also suggest that excessive dentin formation of HGPS is accompanied by odontoblastic activation of dental pulp cells rather than a reaction of odontoblasts in its original place. In addition to its thin impaired dentin, during excessive tertiary dentin formation, a boosting mechanism may exist by soluble factors released by progerin expression. As demonstrated by real-time qPCR using MDPC-23 cells, progerin-expressing odontoblasts secrete a considerable amount of soluble tissue-repairing factors that can stimulate odontogenic differentiation of dental pulp cells. Our *in vitro* results using conditioned media suggest that the paracrine influence of odontoblasts carrying the HGPS mutation gives impetus to the surrounding dental pulp cells to deposit aberrant circumpulpal dentin against dentin thinner than normal coronal dentin as a reparative response.

Taken together, this study shows that the HGPS mutation disturbs physiological secondary dentin formation but also accelerates tertiary dentin formation as a consequence of the progerin expression in odontoblasts. This indicates that progerin expression could be useful to elicit a clinically relevant scenario for human pulp protection through the acceleration of tertiary dentin formation exclusively in aged teeth.

## Materials and Methods

### Generation of HGPS mice

All procedures were performed in accordance with the Karolinska Institutet Guidelines on the Use of Laboratory Animals. All experimental protocols and animal care methods were approved by the Stockholm South Ethical review board, Dnr S141-06, S107-09, S82-12, S101-12 and S24-13. B6.Cg-Tg (*Sp7-tTA, tetO-EGFP/cre*)*1Amc/J* promoter mice (referred to as *Sp7-tTA*) were obtained from the Jackson Laboratory (Bar Harbor, ME, USA) and maintained with drinking water including 100 μg/ml doxycycline/2.5% sucrose. FVB/N-Tg (tetO-LMNA*G608G,-EGFP)*VF1-07Maer* (referred to as *tetop-LA*^*G606G*^) mice express human lamin A and progerin and have been previously described^40^. To target HGPS transgenic expression to odontoblasts, *tetop-LA*^*G606G*^ mice were intercrossed with *Sp7-tTA* transgenic mice as previously described^17^. The offspring were genotyped according to published protocols^40,41^. WT control tissue material was obtained from littermates negative for the transgenes.

### Histology, immunohistochemistry, and histomorphometry

For histological analysis, mice were sacrificed with an overdose of Isofluran (Baxter, Sweden) at postnatal day 5 and postnatal weeks 3, 5, 13 and 20, and the mandibles were carefully dissected. The tissues were fixed in 4% paraformaldehyde (PFA) (pH 7.4) and decalcified in 12.5% ethylenediaminetetraacetic acid (pH 7.0) for three weeks at 4 °C. The decalcified tissues were dehydrated through a graded ethanol series, embedded in paraffin, and sectioned at 5-μm thickness. Slides were stained with hematoxylin and eosin (H&E), and trichrome staining solution (Sigma-Aldrich, St. Louis, MO, USA). For immunohistochemistry, tissue sections were treated with 3% hydrogen peroxide, and incubated with mouse monoclonal anti-human lamin A + C (1:200; MAB3211, Chemicon, Temecula, CA, USA) or rabbit polyclonal antibodies against dentin sialoprotein (Dsp, 1:900; kindly provided by Dr. Larry Fisher) and dentin matrix protein-1 (Dmp1, 1:750; M176, Takara Bio Inc., Shiga, Japan). Histostain Plus Rabbit/Mouse primary (DAB) kits (Zymed laboratories, San Francisco, CA, USA) were used according to the manufacturer’s instructions. Crown dentin thickness was measured in the H&E-stained mid-sagittal sections of mandibular first molars using ANALYSIS software (Soft Imaging System GmbH, Muenster, Germany).

### Micro-CT and scanning electron microscopy (SEM) analysis

For micro-CT analysis, the mandibles were dissected at postnatal week 13, bisected at the symphysis and fixed in 4% PFA. The mandibles were scanned using a desktop scanner (1076 Skyscan Micro-CT, Skyscan, Kontich, Belgium) and analyzed with CTAn software (Skyscan). For SEM analysis, the mandibles were dissected at postnatal week 13 and fixed in 70% ethanol at room temperature for 24 h. The tissue specimens were dehydrated through a graded ethanol series, embedded in methylmethacrylate without decalcification and sectioned through the center of the first mandibular molar using a water-cooled diamond-impregnated circular saw (IsoMet; Buehler, Lake Bluff, IL, USA). The cut surface was polished using 1, 0.3 and 0.05 μm alumina alpha MicroPolish II solutions (Buehler) on a soft cloth rotating wheel. Each sample was placed in an ultrasonic bath between steps and immediately following the polishing steps. The surfaces were acidetched with 37% phosphoric acid for 2–10 seconds, followed by 5% sodium hypochlorite for 20 minutes. The specimen surfaces were sputter-coated with platinum after drying and examined with a scanning electron microscope (JSM-6400; JEOL, Tokyo, Japan) under 20-kV conditions.

### Cell cultures

MDPC-23 cells^42^, a murine dental papilla cell line, were maintained in Dulbecco’s modified Eagle medium (DMEM; Invitrogen, New York, NY, USA) with 10% fetal bovine serum (FBS; Invitrogen), and 100 IU/ml penicillin-100 μg/ml streptomycin (Invitrogen). For mouse dental pulp cells (DPCs), the pulp of mandibular first and second molars from 7- to 8-day-old mice was isolated and digested in a solution of 3 mg/mL collagenase type I (Worthington Biochemical Corp., Freehold, NJ, USA) and 4 mg/mL dispase (Boehringer, Mannheim, Germany) in a serum-free alpha modification of Eagle’s medium (α-MEM; Invitrogen) for 1 h at 37 °C. Single cell suspensions were cultured in a growth medium of α-MEM with 10% FBS, and 100 IU/ml penicillin-100 μg/ml streptomycin (Invitrogen) at 37 °C in 5% CO_2_. Wnt agonist 1 (2-Amino-4-(3,4-(methylenedioxy)benzylamino)-6-(3-methoxyphenyl)pyrimidine, Merck, Darmstadt, Germany), was treated to MDPC-23 cells at the concentration of 10 μM for 16 h, in which cell toxicity was not detected. To induce odontogenic differentiation of mouse dental pulp cells and undifferentiated MDPC-23 cells, conditioned media of MDPC-23 cells expressing WT lamin A and progerin were harvested after treatment of osteogenic medium containing growth media supplemented with 50 μg/ml ascorbic acid (Sigma Aldrich, St. Louis, MO, USA) and 10 mM β-glycerophosphates (Sigma Aldrich) for 48 h. To neutralize the effect of soluble Ctgf, cells were treated with polyclonal rabbit anti-Ctgf IgG or normal rabbit IgG (2 μg/ml) in the conditioned medium for 2 days.

### DNA constructs and transfection

Three expression vectors were generated in the pcDNA3.1 vector. The G608G lamin A expression vector contained a minigene of human lamin A (exons 1–11, intron 11 and exon 12) with the common *LMNA* c.1824C > T HGPS mutation inserted in exon 11. This vector expresses both human lamin A and progerin. The WT lamin A expression vector contained the cDNA of human lamin A and expressed human lamin A. The Δ50 lamin A expression vector contained the cDNA for progerin, including the 150 nucleotide deletion of exon 11, and expressed progerin. Plasmids driving the expression of GFP-tagged human lamin A and the truncated exon 1–12 encoding the cDNA for progerin (Δ50 lamin A) were gifts from Tom Misteli (Addgene plasmids #17662 and #17663, respectively). The TOPflash β-catenin reporter construct containing the Tcf/Lef binding sites and the FOPflash control reporter containing mutated Tcf/Lef binding sites were gifts from Randall Moon (Addgene plasmids #12456 and #12457). The BMP transcriptional reporter (pGL3 BRE luciferase) construct was a gift from Martine Roussel and Peter ten Dijke (Addgene plasmids #45126). The Smad binding element (SBE) reporter (SBE4 luciferase) construct was a gift from Bert Vogelstein (Addgene plasmids #16495). Transfection experiments were performed with Lipofectamine^TM^ LTX and PLUS reagent (Invitrogen) according to the manufacturer’s instructions. After 24 h, transfected cells expressing WT lamin A and progerin were selected with 10 μg/ml puromycin (Santa Cruz Biotechnology, Inc., Dallas, TX, USA) and cultured for further experiments.

### Statistical analysis

Data are presented as the mean ± standard error (SEM) from three or more separate experiments, as indicated. All statistical analyses were done using GraphPad Prism software (GraphPad Software, Inc., La Jolla, CA, USA). Statistical differences were determined by Student’s t test and values of *p* < 0.05 were considered statistically significant.

## Electronic supplementary material

Supplementary Information
