# Supplementary material for: Expression of the Hutchinson-Gilford Progeria Mutation Leads to Aberrant Dentin Formation
Source: Sci Rep. 2018 Oct 18;8:15368. doi: 10.1038/s41598-018-33764-6 (PMC6193977; doi:10.1038/s41598-018-33764-6)

Supplementary Information

Expression of the Hutchinson-Gilford Progeria Mutation Leads to Aberrant Dentin Formation

Hwajung Choi[1,#], Tak-Heun Kim[1,#], Ju-Kyeong Jeong[1], Charlotte Strandgren[2], Maria Eriksson[2], Eui-Sic Cho[1,*]

[1]Cluster for Craniofacial Development and Regeneration Research, Institute of Oral Biosciences, Chonbuk National University School of Dentistry, Jeonju 54896, South Korea

[2]Department of Biosciences and Nutrition, Center for Innovative Medicine, Karolinska Institutet, Huddinge SE-14183, Sweden

Supplementary Table S1. Primer sequences for real-time qPCR

	Sense	Antisense
Osx	TCTCCATCTGCCTGACTCCT	AGCGTATGGCTTCTTTGTGC
Col1a1	CCGGAAGAATACGTATCACC	ACCAGGAGGACCAGGAAGTC
Col1a2	CAGCGAAGAACTCATACAGCC	TTGGAGCAGCCATCGACTA
Oc	ACCCTGGCTGCGCTCTGTCTCT	GATGCGTTTGTAGGCGGTCTTCA
Dmp1	AGTGAGTCATCAGAAGAAAGTCAAGC	CTATACTGGCCTCTGTCGTAGCC
Alpl	AGGGCAATGAGG TCACATCC	GCATCTCGTTATCCGAGTACCAG
Dspp	AACACATCCAGGAACTGCAGCACA	TGACTCGGAGCCATTCCCATCTCT
Phex	GTGGTGGTCTGTGGAATCAG	AGCCGGCTTTCTTCCAATA
Acp4	CTCCTACCCCACAGATCCAC	TCAGAAATCGGCCCAGTTCT
Ctgf	GTGAGTCCTTCCAAAGCAGC	TAGTTGGGTCTGGGCCAAAT
Tgfb1	CCTGCAAGACCATCGACATG	TGTTGTACAAAGCGAGCACC
Tgfb2	GCCCACTTTCTACAGACCCT	CCTTGCTATCGATGTAGCGC
Tgfb3	AGCATCCACTGTCCATGTCA	TTCTTCCTCTGACTGCCCTG
Bmp2	TGCACCAAGATGAACACAGC	GTGCCACGATCCAGTCATTC
Bmp4	CTTCAACCTCAGCAGCATCC	GATGAGGTGTCCAGGAACCA
Il-6	TACCACTCCCAACAGACCTG	ACTCCAGAAGACCAGAGGAA
Il-8	CAGTTTTGCCAAGGAGTGCT	ACTTCTCCACAACCCTCTGC
Mmp3	GTGTGTGGTTGTGTGCTCAT	TTCCCTGTCATCTCCAACCC
Timp1	TTCAAGGCTGTGGGAAATGC	CCACAGCCAGCACTATAGGT
Gapdh	TGCCCAGAACATCATCCCT	GGTCCTCAGTGTAGCCCAAG

Supplementary Methods

Immunofluorescence analysis

Cells were fixed in 4% paraformaldehyde in PBS at room temperature for 10 min. They were then permeabilized by incubation in 0.2% Triton X-100 for 10 min at room temperature, after which endogenous peroxidase activity was quenched by incubation in 3% H_2O_2 in the dark. After rinsing with PBS, nonspecific binding sites on the cells were blocked with 5% BSA in PBS for 30 min at room temperature. Cells were then incubated with primary antibodies against γH2AX (1:250; ab26350, Abcam, Cambridge, MA, USA) and phosphorylated p53 (p-p53; 1:200; sc-21872, Santa Cruz Biotechnology) and active β-catenin (non-phosphorylated S33/37/Thr41; 1:500; 8814, Cell signaling, Danvers, MA, USA) for 16 h at 4°C. Alexa Fluor®-conjugated secondary antibodies (Invitrogen) were used for detection. DAPI was used for counterstaining. Cell staining was evaluated using a fluorescence microscope (Carl Zeiss, Ostalbkreis, Germany).

Luciferase activity

Luciferase activity was determined using the Dual-Luciferase reporter assay system (Promega), according to the manufacturer's instructions. Light intensity was measured with a luminometer, and the luciferase activity was divided by that of the control reporter to normalize for transfection efficiency.

Cell growth analysis

Growth rates of MDPC-23 were measured using the Cell Counting Kit-8 (Dojindo Laboratories, Kumamoto, Japan) according to the manufacturer's instructions. In brief, cells were seeded in 24-multiwell plates and treated with 10 µl/well of the kit solution after 24-h incubation with 24-h interval. Absorbance was measured spectrophotometrically at 450 nm.

Mineralization induction and alizarin red S staining

To induce cell differentiation and mineralized nodule formation of dental pulp cells, 95% confluent cells were cultured in osteogenic media containing α-MEM with 10% FBS, 50 µg/ml ascorbic acid, 10 mM β-glycerophosphates, and 10 nM dexamethasone (Sigma Aldrich) for up to 6 days. Mineral nodule formation was observed by staining the cells with 40 mM alizarin red S (pH 4.2) after fixation with 4% PFA for 10 min. The amount of alizarin red S that bound to the minerals was quantified by destaining the samples in 10 mM sodium phosphate containing 10% cetylpyridinium chloride (pH 7.0) for 15 min at room temperature. The amount of alizarin red S in the destaining solution was measured at OD 562 nm.

RNA preparation and real-time qPCR

1.0 x 10^5 cells were seeded on a 35mm culture dish, transfected next day for 24 h and cultured with new media for additional 24 h before RNA preparation. For paracrine experiments, cells were treated for 2 days with conditioned media harvested from MDPC-23 cells transfected with Δ50 lamin A, WT lamin A and the negative control. Total RNA was prepared using an RNeasy Mini kit (QIAGEN, Valencia, CA, USA) according to the manufacturer's instructions, and cDNA was synthesized from 2 µg of total RNA using Superscript II reverse transcriptase (Invitrogen). Real-time PCR was performed with SYBR

Green PCR Master Mix (Applied Biosystems, Warrington, Cheshire, UK) following the manufacturer's protocols. Reaction conditions comprised 40 cycles of 15 sec of denaturation at 95℃ and 1 min of amplification at 60℃. All reactions were run in triplicate, and expression was normalized to that of the housekeeping gene *glyceraldehyde-3-phosphate dehydrogenase* (*Gapdh*). Relative levels of transcript expression were quantified using the ΔΔ*Ct* method. The calculation was performed using the *Ct* value of *Gapdh* to normalize the *Ct* value of the target gene in each sample and obtain the Δ*Ct* value, which then was used to compare different samples. Relative mRNA expression was compared in a histogram. Specific primers sets used in the analysis are listed in Supplementary Table S1.

Western blot analysis

Proteins (30 - 100 μg) were dissolved in sample buffer, and electrophoresis was carried out at a current of 25 mA for 2 h. Proteins were transferred from SDS-PAGE onto nitrocellulose membranes (Schleicher & Schuell, Dassel, Germany). Membranes were blocked for 1 h with 5% nonfat dry milk in PBS containing 0.1% Tween-20 (PBS-T) and incubated overnight with human lamin A+C (1:25; MAB3211, Chemicon), γH2AX (1:500; ab26350, Abcam), phosphor-p53 (p-p53; 1:100, sc-21872, Santa Cruz Biotechnology), active β-catenin (non-phosphorylated S33/37/Thr41; 1:2000; 8814, Cell signaling), β-catenin (1:2000; RB-9035, Thermo Scientific), histone H3 (1:2000; ab1791, Abcam), α-tubulin (1:1000; sc-8035, Santa Cruz Biotechnology), Ctgf (1:1000; ab6992, abcam) and β-Actin (1:2000, sc-1616, Santa Cruz Biotechnology) IgG diluted in PBS-T buffer at 4 ℃. After washing, the membranes were incubated with anti-rabbit or mouse-IgG conjugated horseradish peroxidase (Santa Cruz Biotechnology) for 1 h. Labeled protein bands were detected using an enhanced

chemiluminescence system (Amersham Biosciences, Buckinghamshire, UK). Protein expression levels were analyzed with the ImageQuanT TL 1D gel analysis program (Amersham Biosciences).

Legends for Supplementary Figures

Supplementary Figure S1. Immunofluorescence staining of phosphorylated p53 in MDPC-23 with Δ50 lamin A. Phosphorylated p53-positive MDPC-23 cells with Δ50 lamin A were detected by using an antibody for phosphor-p53 (p-p53) (a) and their counted percentage (b) compared to the WT lamin A and the negative control. Data are presented as mean ± SEM of three measurements in each group. *Bars*, 5 μm.

Supplementary Figure S2. Immunofluorescence staining of non-phosphorylated active β-catenin. The impaired nuclear translocation of active β-catenin was visualized by immunofluorescence staining using an antibody for non-phosphorylated β-catenin (S33/37/Thr41) after treatment with and without Wnt agonist 1 compared to the WT lamin A and the negative control. *Bars*, 5 μm.

Supplementary Figure S3. Transcript levels of BMPs and genes associated with inflammation in progerin-expressing odontoblasts. The mRNA expression levels of BMPs and genes associated with inflammation were analyzed by real-time qPCR. RNA was isolated from MDPC-23 transfected with Δ50 lamin A, WT lamin A and the negative control. Data are presented as mean ± SEM of three measurements in each group.

Supplementary Figure S4. Soluble factors induced by progerin induce the differentiation of pre-odontoblasts. (a) Mineralization ability of MDPC-23 cells treated with conditioned media from MDPC-23 cells transfected with Δ50 lamin A (Δ50-CM), WT

lamin A (WT-CM) and the negative control (Control-CM) was exhibited by alizarin red staining and evaluated. (b) The transcript levels of odontogenesis-associated genes were analyzed by real-time qPCR with MDPC-23 cells treated with conditioned media of Δ50 lamin A (Δ50-CM), WT lamin A (WT-CM) and the negative control (Control-CM) for 2 days. Significance was assigned for *p*-values as indicated.

Supplementary Figure S5. Original full-size blots of Figure 3E. The following antibodies were used: human lamin A+C (Human LA; 1:25; MAB3211, Chemicon), γH2AX (1:500; ab26350, Abcam), phosphorylated p53 (p-p53; 1:100, sc-21872, Santa Cruz Biotechnology) and β-Actin (1:2000, sc-1616R, Santa Cruz Biotechnology).

Supplementary Figure S6. Original full-size blots of Figure 4C. The following antibodies were used: active β-catenin (non-phosphorylated S33/37/Thr41; 1:2000; 8814, Cell signaling), β-catenin (1:2000; RB-9035, Thermo Scientific), histone H3 (1:2000; ab1791, Abcam), and α-tubulin (1:1000; sc-8035, Santa Cruz Biotechnology).

Supplementary Figure S7. Original full-size blots of Figure 6A. The following antibodies were used: Ctgf (1:1000; ab6992, abcam) and β-Actin (1:2000, sc-1616, Santa Cruz Biotechnology).

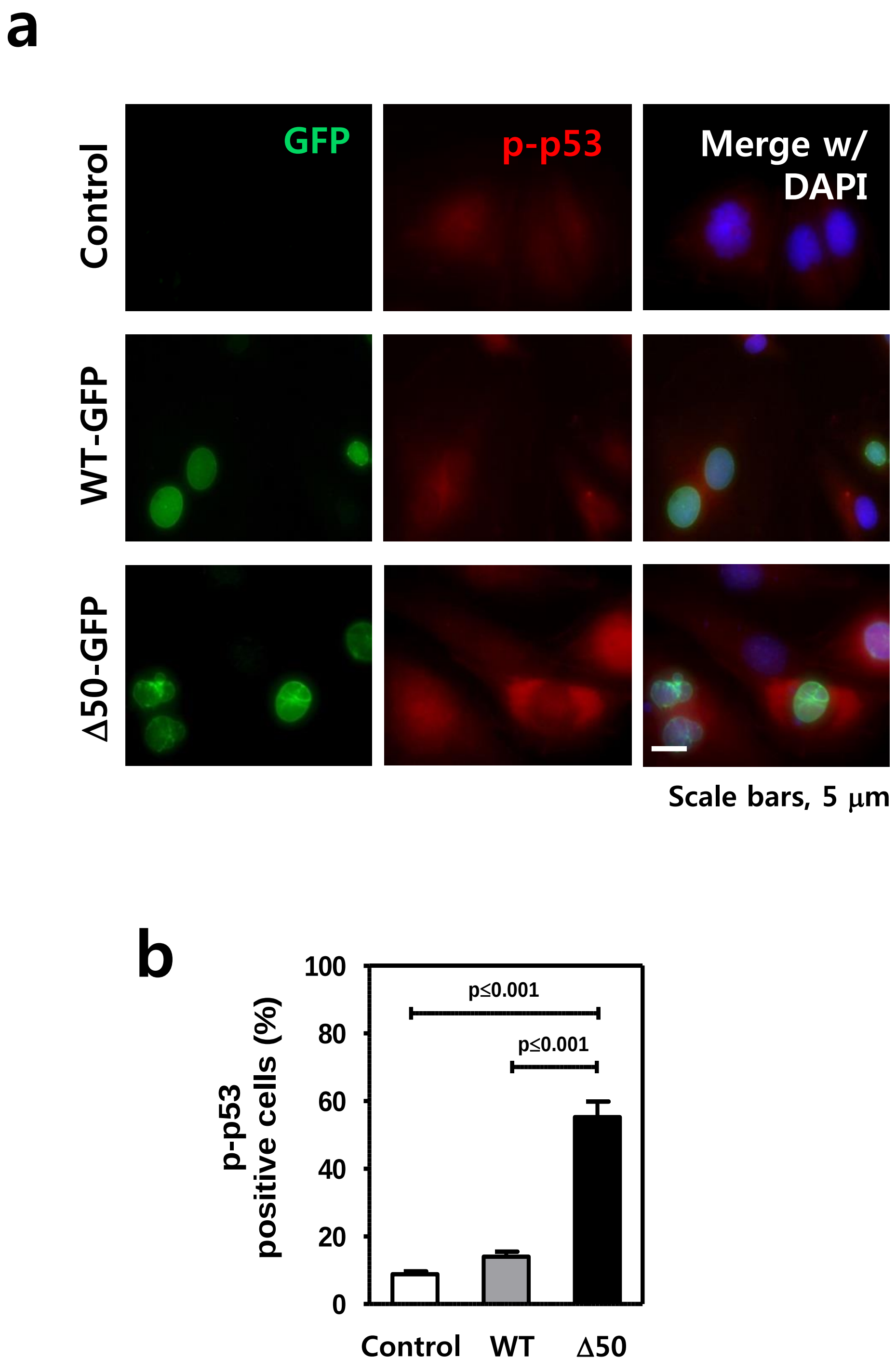
a
GFP
p-p53
Merge w/
DAPI
Control
WT-GFP
Δ50-GFP
Scale bars, 5 μm
b
p-p53
positive cells (%)
100
80
60
40
20
0
p≤0.001
p≤0.001
Control
WT
Δ50

Supplementary Figure S2

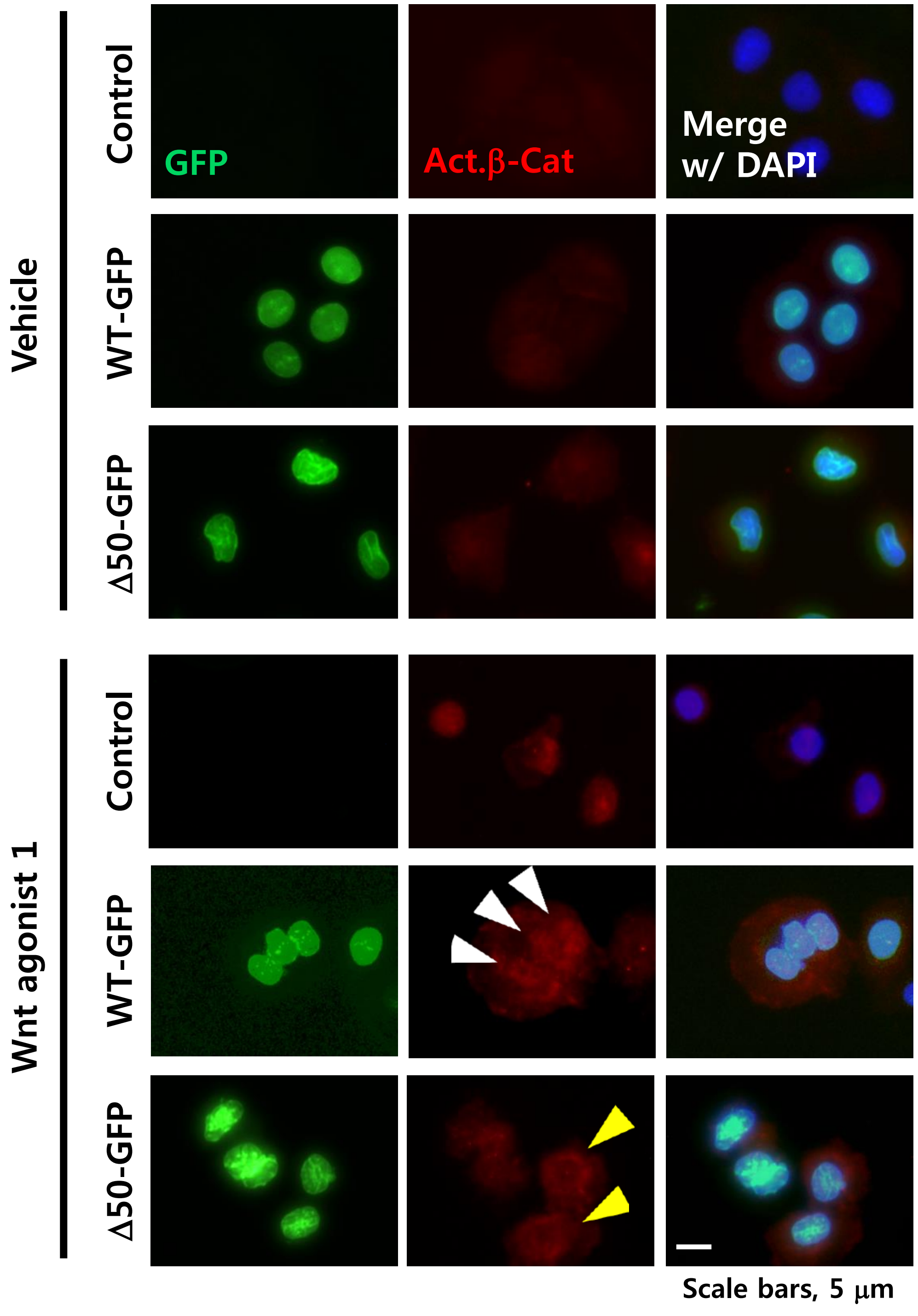

Supplementary Figure S3

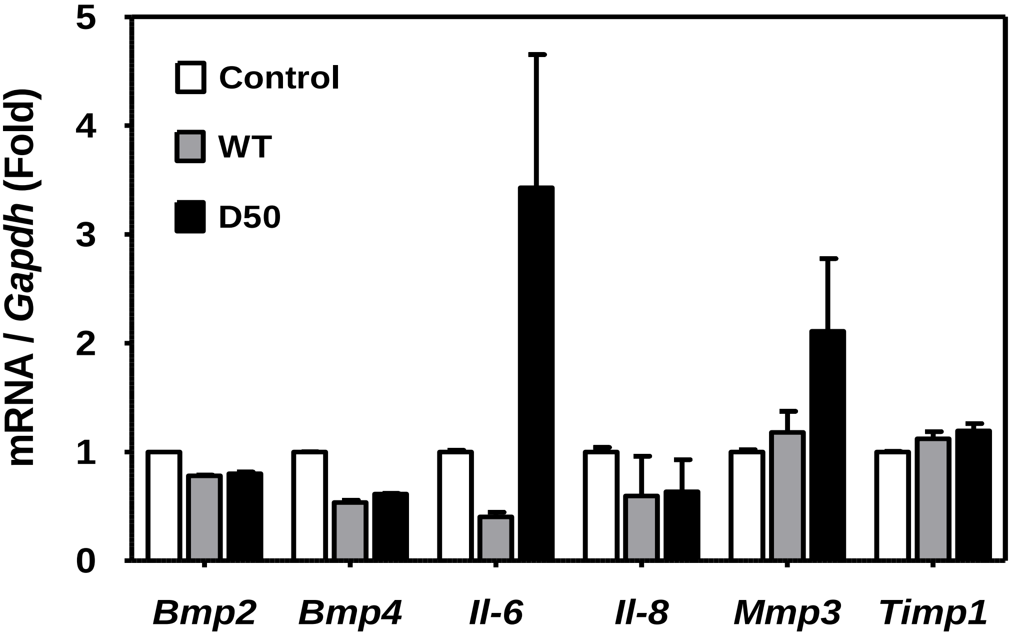

a

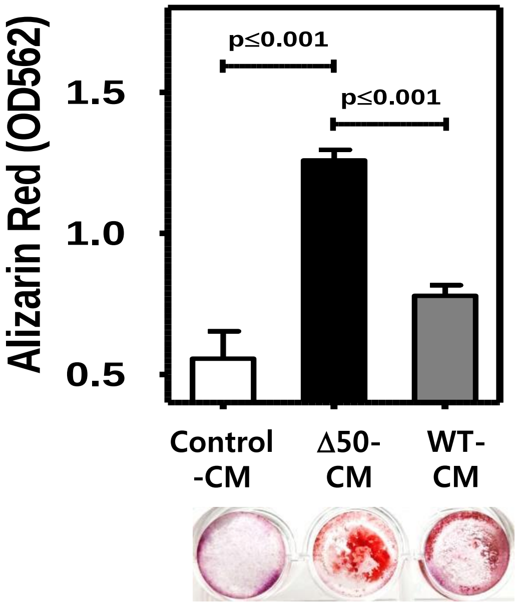
p≤0.001
p≤0.001
Alizarin Red (OD562)
1.5
1.0
0.5
Control -CM
Δ50- CM
WT- CM

b

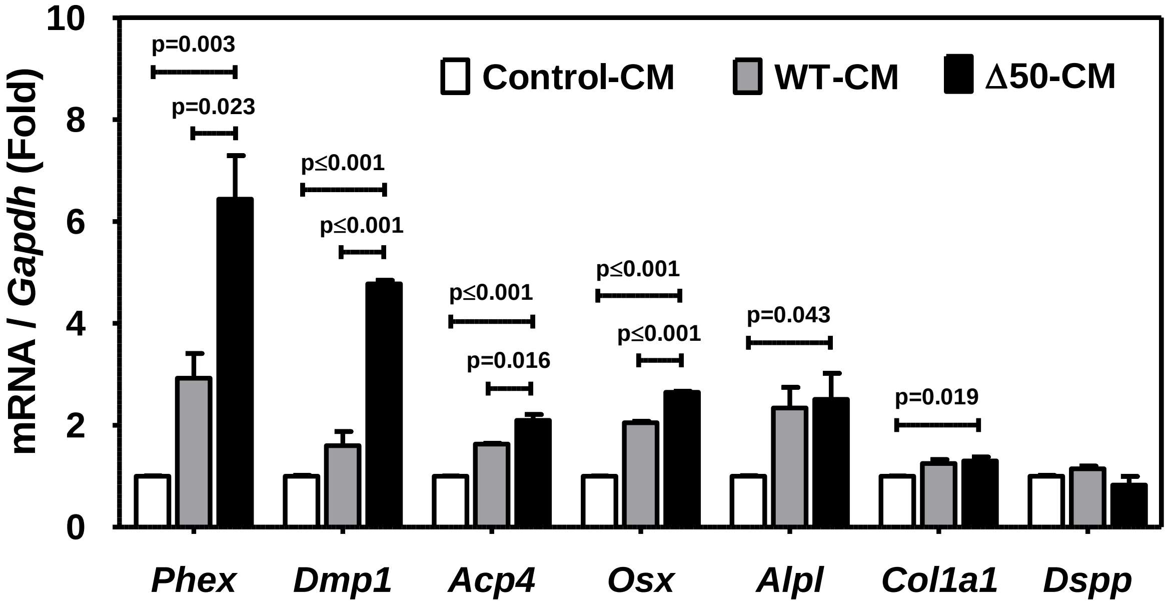
Control-CM
WT-CM
Δ50-CM
mRNA / Gapdh (Fold)
10
8
6
4
2
0
p=0.003
p=0.023
p≤0.001
p≤0.001
p≤0.001
p=0.016
p≤0.001
p≤0.001
p=0.043
p=0.019
Phex
Dmp1
Acp4
Osx
Alpl
Col1a1
Dspp

Supplementary Figure S5

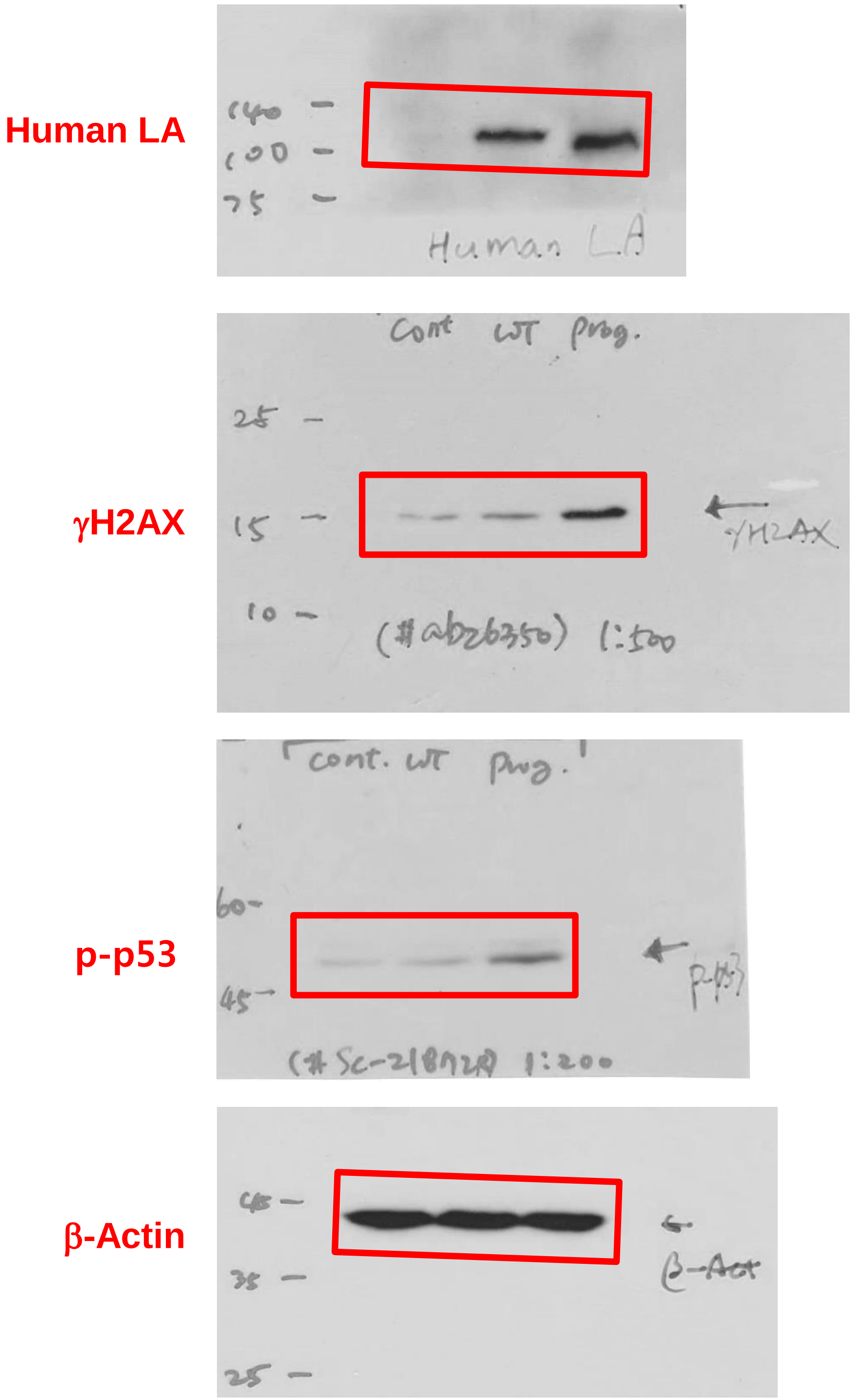

Supplementary Figure S6

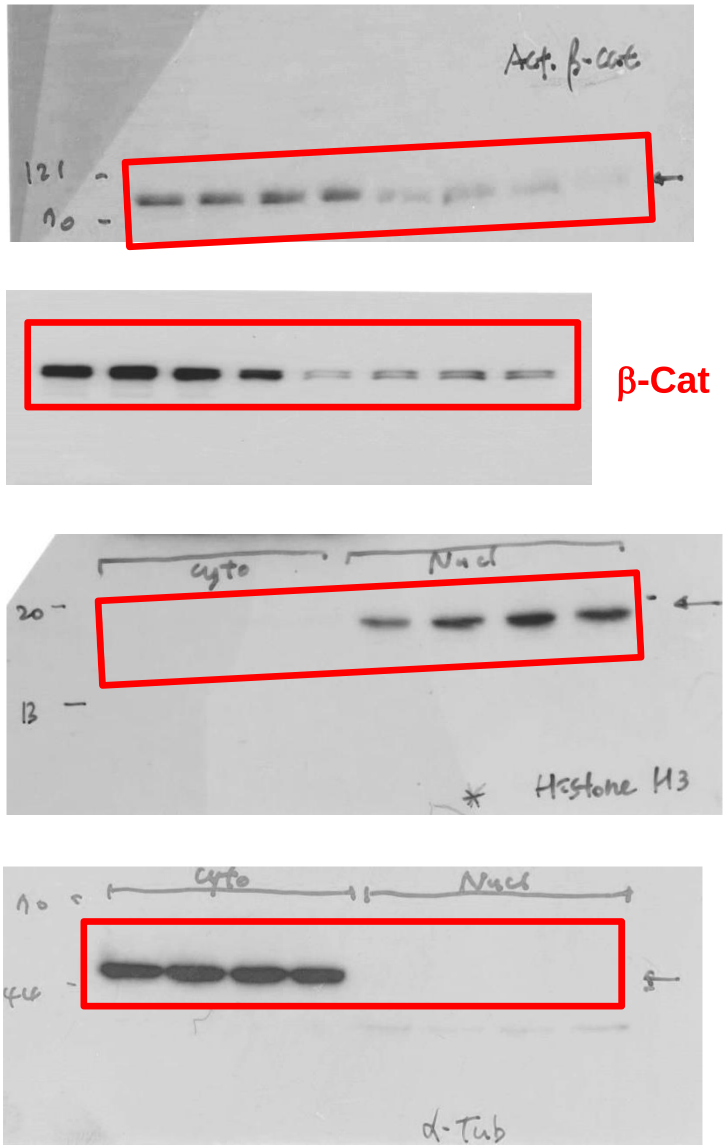

Active β-Cat

β-Cat

Histone H3

α-Tubulin

Supplementary Figure S7

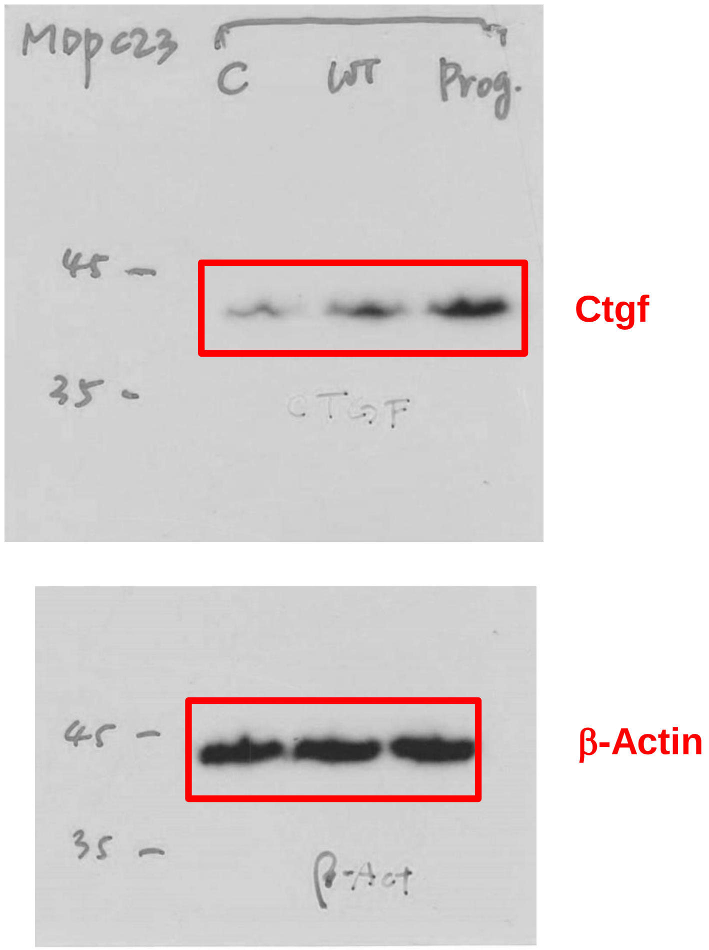

Supplement: Supplementary file 1 — Supplementary Information [file 41598_2018_33764_MOESM1_ESM.pdf]
